# Wheezing Sound Separation Based on Informed Inter-Segment Non-Negative Matrix Partial Co-Factorization

**DOI:** 10.3390/s20092679

**Published:** 2020-05-08

**Authors:** Juan De La Torre Cruz, Francisco Jesús Cañadas Quesada, Nicolás Ruiz Reyes, Pedro Vera Candeas, Julio José Carabias Orti

**Affiliations:** Departament of Telecommunication Engineering, University of Jaen, Campus Cientifico-Tecnologico de Linares, Avda. de la Universidad, s/n, 23700 Linares, Jaen, Spain; fcanadas@ujaen.es (F.J.C.Q.); nicolas@ujaen.es (N.R.R.); pvera@ujaen.es (P.V.C.); carabias@ujaen.es (J.J.C.O.)

**Keywords:** sound separation, non-negative matrix partial co-factorization, bases, repetitive, sharing, wheezing, normal respiratory sounds, informed, inter-segment

## Abstract

Wheezing reveals important cues that can be useful in alerting about respiratory disorders, such as Chronic Obstructive Pulmonary Disease. Early detection of wheezing through auscultation will allow the physician to be aware of the existence of the respiratory disorder in its early stage, thus minimizing the damage the disorder can cause to the subject, especially in low-income and middle-income countries. The proposed method presents an extended version of Non-negative Matrix Partial Co-Factorization (NMPCF) that eliminates most of the acoustic interference caused by normal respiratory sounds while preserving the wheezing content needed by the physician to make a reliable diagnosis of the subject’s airway status. This extension, called Informed Inter-Segment NMPCF (IIS-NMPCF), attempts to overcome the drawback of the conventional NMPCF that treats all segments of the spectrogram equally, adding greater importance for signal reconstruction of repetitive sound events to those segments where wheezing sounds have not been detected. Specifically, IIS-NMPCF is based on a bases sharing process in which inter-segment information, informed by a wheezing detection system, is incorporated into the factorization to reconstruct a more accurate modelling of normal respiratory sounds. Results demonstrate the significant improvement obtained in the wheezing sound quality by IIS-NMPCF compared to the conventional NMPCF for all the Signal-to-Noise Ratio (SNR) scenarios evaluated, specifically, an SDR, SIR and SAR improvement equals 5.8 dB, 4.9 dB and 7.5 dB evaluating a noisy scenario with SNR = −5 dB.

## 1. Introduction

Chronic Respiratory Diseases (CRDs) can be defined as disorders of the airways and other physiological structures of the respiratory system. One of the most common CRDs is Chronic Obstructive Pulmonary Disease (COPD) that is responsible for more than 3 million deaths of people each year which is equivalent to 6% of all deaths worldwide [1]. COPD is often characterized by the presence of wheeze sounds since wheezes provide relevant clues that alert about a respiratory disorder [2,3]. Although CRDs currently have no medical cure, early detection of wheezing from auscultation can lead to treatment when the disease is in its early stage, thus improving people’s quality of life. Although there are other clinical alternatives, such as chest radiography and laboratory analysis, auscultation remains the main technique used in most of the health centers in low-income and middle-income countries to provide the first medical diagnosis of the status of the lung due to its low cost, safety and non-invasive nature. Nevertheless, this early detection by the physician depends largely on the subjective diagnosis based on both the training and expertise in interpreting what hears with the stethoscope and the vulnerability to normal respiratory sounds that can mask the presence of sounds of interest, such as wheezing [4]. Today, many researchers continue to investigate in biomedical signal processing to enhance the clarity of the wheezing sounds with the aim that all useful medical information contained in the wheezing sound signal is heard in the process of auscultation.

**Figure 1:**
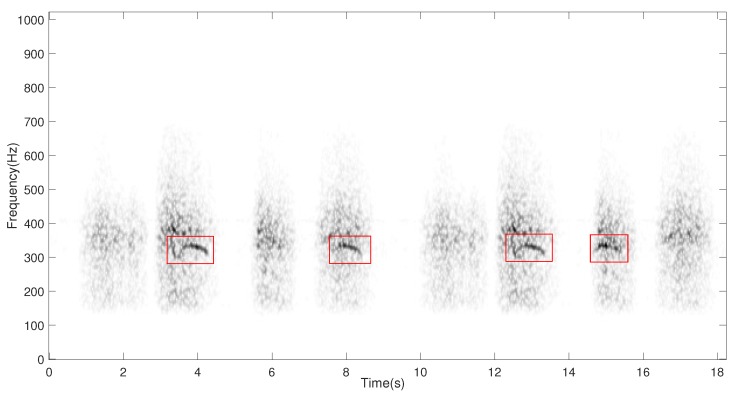
Time-frequency representation of a breathing recording from an unhealthy subject in which four wheezes (red rectangles), mixed with normal respiratory sounds, can be observed. Higher energies are indicated by darker colour.

In general terms, the respiratory sounds can be classified into two main categories: normal and abnormal (adventitious, such as wheezes), according to the Computerized Respiratory Sound Analysis (CORSA) guidelines [5]. Although wheeze and normal respiratory sounds appear simultaneously since both of them are generated by the same air flow through the lungs, normal respiratory sounds are always present in each respiratory cycle since they are automatically generated by the breathing process. However, the occurrence of wheezing sounds is random because of the respiratory disorder so they do not have to be present in all breathing cycles. So, normal respiratory sounds (RS) are generated by healthy lungs and they are represented by broadband spectrum where most of the energy is concentrated in the spectral band 60 Hz–1000 Hz [6]. Wheeze sounds (WS) are abnormal sounds, generated by unhealthy lungs that suffer narrowing of airways, superimposed onto the RS. Therefore, WS can be described as pitched and continuous sounds which usually have a fundamental frequency (pitch) located between 100 Hz–1000 Hz with duration longer than 100 ms, displaying spectral trajectories of narrowband spectral peaks [7] as shown in Figure 1. In this work, any single-channel signal composed of both RS and WS will be referred as mixture.

It is common that the cognitive capacity of the physician is reduced throughout the day as the number of hours spent analyzing respiratory sounds increases, a fact that is exacerbated by the stress to which the physician is subjected to certain medical cases [8,9]. The presence of WS is often associated with obstructions of the airways. However, the interference caused by RS causes the loss of relevant wheezing content in WS which makes it difficult to provide a reliable diagnosis of the status of the lung according to what is being heard through the stethoscope. Sound source separation approaches have been widely applied to overcome this problem by isolating the sounds of interest (target) from those that act as acoustic interference (non-target) [10].

Many biomedical signal processing challenges, such as ambient denoising [11], wheezing detection and classification are still open to the machine learning research community. In [11], a denoising approach is proposed to remove ambient noise from lung sound recordings by means of an adaptive subtraction method that operates in the spectral domain. Focusing on both wheezing detection and classification tasks, the initial works are based on spectral peaks analysis applying thresholding [2,12,13,14,15] that obtain sensitivity/specificity results from 71% to 98%. Like this, Taplidou and Hadjileontiadis [14] proposed a spectro-temporal wheeze detector that automatically locates and identifies wheeze sounds based on spectral trend elimination, separation of the spectrum into frequency bands and peak detection/classification. Most of the wheezing detection and classification approaches are based on the feature extraction and classifier configuration: (i) Musical features and Logistic Regression Model (LRM) [16]; (ii) Spectral features and Support Vector Machine (SVM) such as Power spectral density mean and harmonics [17], Intensity, mean frequency and standard deviation frequency [18], Power spectral band [19], Tonality index [20] and Ensemble Empirical Mode Decomposition (EEMD) [21]; and finally, (iii) Mel Frequency Cepstral Coefficients (MFCC) using K-nearest neighbour (KNN) [22], LRM [23] and Gaussian Mixture Model (GMM) [24], that obtain sensitivity/specificity results from 90% to 99%. Thus, a wheezing detection [20] was developed at the segment level by means of a SVM classifier whose features are the spectral envelope variation and a tonality index. Other works have been focused on the wavelet domain [25,26]. In this context, Ulukaya et al. [26] presented a tunable RAtional Dilation Wavelet Transform (RADWT) based method to discriminate monophonic and polyphonic wheeze sounds by means of localized energy peaks which are calculated from wavelet coefficients. Other studies have applied different types of neural networks (NN) to wheezing sound analysis [27,28,29,30] obtaining the best promising performance in terms of sensitivity and specificity results, specifically, from 86% and 100%. Thus, Lin et al. [27] introduce a method that searches for horizontal or nearly horizontal edges of the spectrogram and a back-propagation neural network (BPNN) classifier is applied using features such as, frequency range and the slope of the potential wheeze. However, wheezing detection and classification tasks could be improved applying sound source separation techniques as a preliminary step since these techniques can increase the clarity of the wheezing content hidden in the signal being auscultated. Although very few works [31,32] have addressed in depth the separation of wheezing sound sources to the best of our knowledge, all of them are based on Non-negative Matrix Factorization (NMF) since NMF is a recent and promising tool that can extract hidden sound events with physical interpretation in nature. Specifically, Torre et al. [31] present a constrained NMF approach to separate wheezes from respiratory sounds applied to single-channel mixtures. The proposed constraints, smoothness and sparseness, model common spectral behavior shown by wheezes and normal breath sounds. Results report that the proposed method improves the acoustic quality of the wheezes removing most of the respiratory sounds.

In this paper, an extended version of Non-negative Matrix Partial Co-Factorization (NMPCF) is proposed to suppress RS while preserving the wheezing acoustic content. Here, we assume that RS can be considered as repetitive sound events during breathing so, RS can be modeled by sharing together the spectral patterns found in each respiratory stage (segment), inspiration or expiration, with a respiratory training signal. However, this sharing of patterns can not be applied to wheezes since WS could not be present at each segment due to their unpredictable nature in time motivated by the pulmonary disorder. To improve the sound separation performance of the conventional NMPCF that treats equally all segments of the spectrogram, the main contribution of the proposed method adds higher importance to those segments classified as non-wheezing using inter-segment information informed by a wheezing detection system. As a result, our proposal is able to characterize RS more accurately by forcing to model more on those non-wheezing segments in the bases sharing process into the NMPCF decomposition.

The rest of this paper is structured as follows. First, Section 2 briefly reviews the background of the most relevant approaches based on Non-negative Matrix Factorization and Non-negative Matrix Partial Co-Factorization. Section 3 details the proposed method. Section 4 discusses the evaluation and the experimental results. Finally, conclusions and further research are presented in Section 5.

## 2. Background

### 2.1. Non-Negative Matrix Factorization

Non-negative Matrix Factorization (NMF) [33,34] is a rank-reduction method that has been widely applied to learning images [35] and audio [36]. NMF includes the non-negativity constraint to recover hidden patterns of the input data using basis and activation matrices. Considering a monaural input mixture x(t), composed of sources of interest (target) $x_{W}\left(t\right)$ and non-target sources $x_{R}\left(t\right)$, NMF factorizes the input spectrogram X into the product of two non-negative matrices: basis matrix $U\in R_{+}^{F\times K}$ and activation matrix $V\in R_{+}^{K\times T}$ as shown in Equation (1). We assume an approximate linear additivity between the input spectrograms $X_{W}\in R_{+}^{F\times T}$ and $X_{R}\in R_{+}^{F\times T}$. The subscript *W* is often used to refer the sounds of interest and the subscript *R* is applied to the sounds that act as acoustic interference,

(1) $$X=X_{W}+X_{R}\approx \hat{X}={\hat{X}}_{W}+{\hat{X}}_{R}=UV=\left[\begin{array}{cc} U_{W} & U_{R} \end{array}\right]\left[\begin{array}{c} V_{W}\\ V_{R} \end{array}\right]=U_{W}V_{W}+U_{R}V_{R}$$

obtaining the estimated spectrograms $\hat{X}\in R_{+}^{F\times T}$, ${\hat{X}}_{W}\in R_{+}^{F\times T}$, ${\hat{X}}_{R}\in R_{+}^{F\times T}$ with *F* frequency bins and *T* frames using *K* bases and the corresponding time-varying activations. Therefore, U can be interpreted as a dictionary of spectral bases or patterns that represents the frequency information associated to the target and non-target sources active in the input spectrogram. Instead, V represents a matrix of activations that indicates the activity of each spectral basis in a given frame.

NMF is often calculated using an iterative algorithm, based on multiplicative update rules [33], to obtain those parameters that reduce the cost function $D(X|\hat{X})$ based on penalizing the error reconstruction between X and $\hat{X}$. In this paper, the generalized Kullback-Liebler divergence $D_{KL}\left(X|\hat{X}\right)$ [37] has been applied because it confirms the non-negativity of U and V as can be observed in Equations (3) and (4). In addition, recent works [32,38] report that $D_{KL}\left(X|\hat{X}\right)$ can be used in biomedical signal processing to achieve promising results,

(2) $$D_{KL}\left(X|\hat{X}\right)=X\log\frac{X}{\hat{X}}-X+\hat{X}$$

(3) $$U_{z}\leftarrow U_{z}⊙\left(\left(X⊘\mathrm{UV}\right){V_{z}}^{T}⊘(1V_{z}^{T}\right)),\;z=W,R$$

(4) $$V_{z}\leftarrow V_{z}⊙\left({U_{z}}^{T}\left(X⊘\mathrm{UV}\right)⊘({U_{z}}^{T}1\right)),\;z=W,R$$

where $U_{W}\in R_{+}^{F\times K_{W}}$, $U_{R}\in R_{+}^{F\times K_{R}}$, $V_{W}\in R_{+}^{K_{W}\times T}$ and $V_{R}\in R_{+}^{K_{R}\times T}$ are initialized as random positive matrices, $1\in R_{+}^{F\times T}$ represents an all-ones matrix, ${}^{T}$ is the transpose operator, ⊙ is the element-wise multiplication, ⊘ is the element-wise division and $K=K_{W}+K_{R}$ indicates the number of bases, being $K_{W}$ the number of bases related to the sounds of interest and $K_{R}$ the number of bases related to the acoustic interference.

The main drawbacks shown by NMF can be summarized in the following three points: (i) poor signal quality when the iterative algorithm reaches a poor local minimum; (ii) NMF can not reconstruct each source because it does not have enough information to cluster all the bases generated by the same source; (iii) NMF does not guarantee a parts-based objects reconstruction with physical meaning as occurs in nature [39]. To overcome this problem, three approaches have been widely proposed in literature [40]: (i) supervised NMF (SNMF) [41,42] in which $U_{W}$ and $U_{R}$ are learned in advanced by means of training and fixed during the iterative process. As a result, only the activations matrices $V_{W}$ and $V_{R}$ are updated; (ii) semi-supervised NMF (SSNMF) [43,44] in which $U_{R}$ is learned in advanced by means of training and fixed during the iterative process. As a result, $V_{W}$, $V_{R}$ and $U_{W}$ are updated; and (iii) constrained NMF (CNMF) in which no training is used because different constraints are included into the factorization procedure to model the specific time-frequency characteristics of the sources to extract [45,46].

To sum up, SNMF, SSNMF and CNMF find better solutions compared to NMF since all of them model, into the bases or activations obtained from the factorization, temporal or spectral behaviors shown by the sounds, that are intended to be recovered, in nature. Nevertheless, the main disadvantages observed in both SNMF and SSNMF are the following: (i) highly dependent of the training data so, the separation performance is limited to the spectral similarity between the training and sounds contained in the input mixture and; (ii) there may not be public training databases available. On the other hand, the main disadvantage observed in constrained NMF approaches, such as CNMF is the difficulty of mathematically defining both the constraints that correctly model the temporal and spectral behaviors shown by the target sources and their incorporation into the cost function on which the factorization is based [47].

### 2.2. Non-Negative Matrix Partial Co-Factorization

Non-negative Matrix Partial Co-Factorization (NMPCF) has been used in several audio processing tasks, such as extraction of rhythmic sources [48,49,50], singing-voice separation [51] or speaker diarization [52]. The main idea of NMPCF is to apply a joint matrix factorization using multiple input matrices to obtain a set of shared spectral bases or temporal activations.

In general, NMPCF-based methods can be classified into four approaches: (i) semi-supervised factorization (1S-NMPCF) [50] in which a joint decomposition, considering the input mixture and a training matrix related to repetitive sounds, is performed by sharing some bases active in both of them [48]; (ii) supervised factorization (2S-NMPCF) in which a joint decomposition, considering the input mixture and two training matrices related to repetitive and non-repetitive sounds, is performed by sharing some bases active between each training matrix and the input mixture [51]; (iii) unsupervised factorization (T-NMPCF) [50] in which a joint decomposition using multiple shorter segments from the input mixture is obtained factorizing them into repetitive sound events by finding common bases across segments [49]; and (iv) semi-supervised factorization (ST-NMPCF) [50] in which a joint decomposition of the input mixture is performed using a training matrix associated to repetitive sound events and multiple shorter segments to make advantage of both spectral and temporal modelling of repetitive sounds.

However, NMPCF-based approaches treat all segments of the input mixture decomposition together equally, ignoring the importance of each specific segment in the modelling of the repetitive and non-repetitive sounds. As a result, it could be interesting to investigate how to include the importance of different segments according their spectral content to weight the spectral modelling of the repetitive sounds in the joint factorization and as a consequence, to improve the separation quality of the sounds of interest.

## 3. Proposed Method

**Figure 2:**
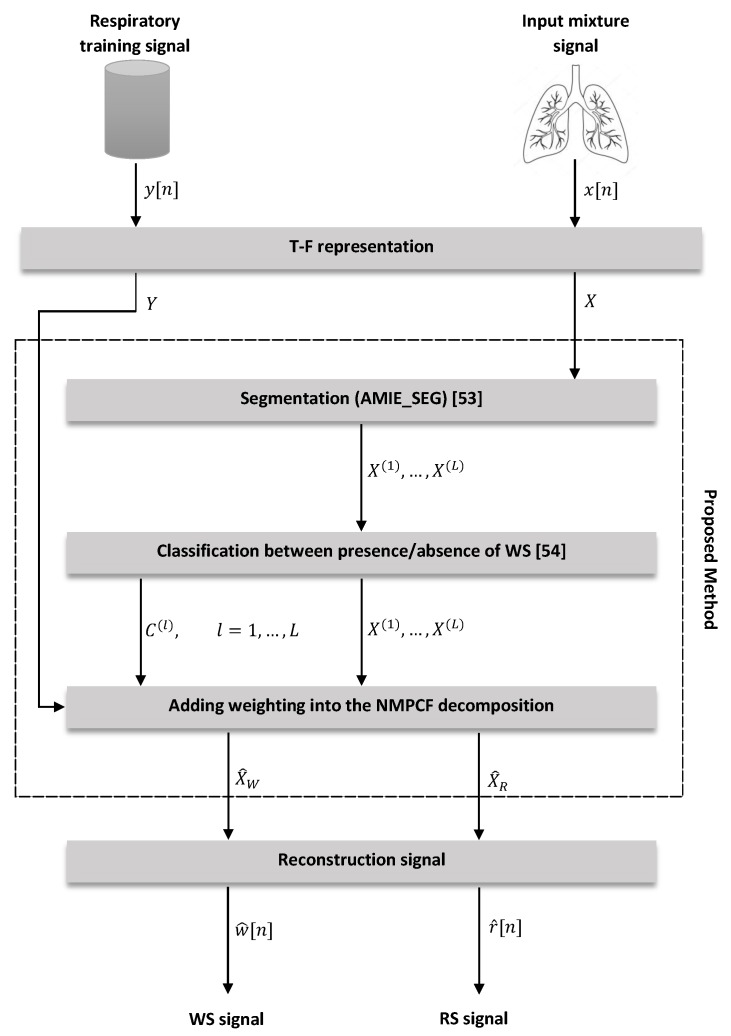
Flowchart of the proposed method IIS-NMPCF.

The aim of the proposed method is to enhance the quality of the WS by removing the RS that implicitly appear in the human breathing process. In order to improve the separation performance between WS and RS of the NMF-based and NMPCF-based baseline methods, we propose a modified NMPCF approach denominated Informed Inter-Segment Non-negative Matrix Partial Co-Factorization (IIS-NMPCF) that adds higher importance into the NMPCF factorization to those segments in which WS are not present. For this purpose, IIS-NMPCF consists of three stages: (i) Segmentation; (ii) Classification between presence/absence of WS and finally (iii) Adding weighting into the NMPCF decomposition. The flowchart of the proposed method is shown in Figure 2, and details are depicted in the following Section 3.1 and Section 3.2.

### 3.1. Time-Frequency Signal Representation

Let x[n] denote the n-th sample of a mixture signal, which consists of the sum of wheezing w[n] and normal respiratory sounds r[n]. The magnitude spectrogram X of a mixture signal x[n] can be represented as $X=X_{W}+X_{R}$, being $X_{W}$ the magnitude spectrogram of only WS and $X_{R}$ the magnitude spectrogram of only RS. Each unit $X_{f,t}$ is defined by the f-th frequency bin at the *t*-th frame and is calculated from the magnitude of the Short-Time Fourier Transform (STFT) using a Hamming window of N samples with 25% overlap. A normalization process is applied in order to ensure that the proposed method can be independent of the size and scale of the magnitude spectrogram X. To avoid complex nomenclature throughout the paper, the variable X is hereinafter referred to the normalized magnitude spectrogram $\bar{X}$ computed as follows,

(5) $$\bar{X}=\frac{X}{\left(\frac{\sum_{f,t}X_{f,t}}{FT}\right)}$$

Besides, y[n] denote the n-th sample of the respiratory training signal, which consists of a concatenation of different respiratory stages composed only of RS (for more details see Section 4.3). The magnitude spectrogram Y of the respiratory training signal y[n] has been calculated following the same procedure used with the previous magnitude spectrogram X.

### 3.2. Wheezing Sound Separation Using Informed Inter-Segment NMPCF

The key assumptions behind the proposed method IIS-NMPCF to apply WS and RS source sound separation are the following:

(i) RS are often characterized by similar spectral patterns that represent a wideband noise spectrum showing time and frequency smoothness [32]. In this way, Y can be useful to replicate these similar RS spectro-temporal behaviors observed in most of the subjects.
(ii) In addition, RS can be considered as repetitive events in human breathing so, RS can be modeled sharing common spectral patterns that can be found throughout all breathing stages (segments), that is, some basis vectors can be shared during the inter-segment analysis due to the repeatability of RS. If we divide the input mixture spectrogram X into segments $X^{\left(1\right)}$,$X^{\left(2\right)}$, …,$X^{\left(L\right)}$, we can get *L*-segments from the given mixture x[n] that share common spectral patterns. For this purpose, we have used AMIE_SEG [53] that automatically allows to segment the mixture spectrogram X into inspiratory and expiratory stages.
(iii) However, WS can be present or absent in the respiratory stages due to the pulmonary disorder. Therefore, we can define an indicator $C^{\left(l\right)}$ to distinguish between non-wheezing ($C^{\left(l\right)}=0$) and wheezing ($C^{\left(l\right)}=1$) segments. Note that the term ${}^{\left(l\right)}$ refers to the segment identifier l=1,…,L of the mixture spectrogram X. In the case of wheezing segments, the spectral patterns of both RS and WS are present. For this reason, we propose to weight the importance of wheezing and non-wheezing segments into the conventional NMPCF decomposition to improve the wheezing sound separation performance. The classification between non-wheezing and wheezing segments is provided by a wheezing detection algorithm previously developed by authors [54].

**Figure 3:**
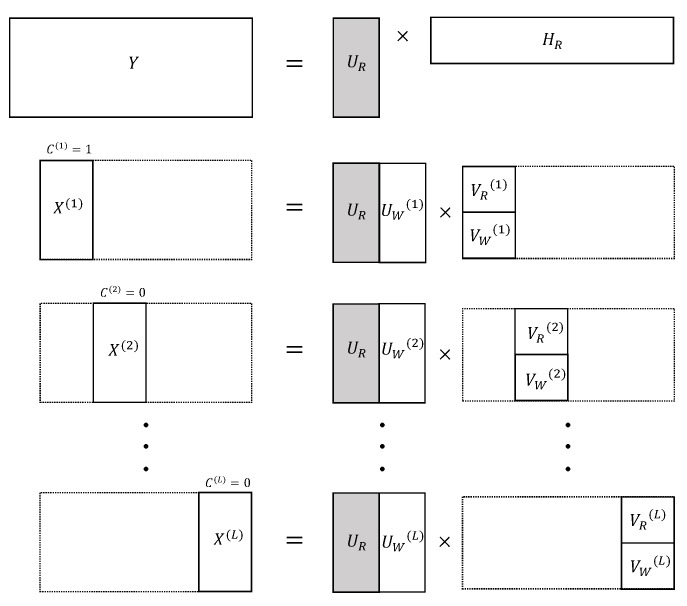
Pictorial illustration of the matrix decomposition based on IIS-NMPCF.

Considering two input spectrograms X and Y, the factorization of the conventional ST-NMPCF lets the common respiratory basis vectors $U_{R}$ be shared jointly between the spectrogram Y and *L*-segments $X^{\left(1\right)}$,$X^{\left(2\right)}$, …, $X^{\left(L\right)}$ of the input spectrogram X (see Figure 3),

(6) $$X^{\left(l\right)}\approx {\hat{X}}^{\left(l\right)}={\hat{X}}_{R}^{\left(l\right)}+{\hat{X}}_{W}^{\left(l\right)}=U_{R}diag\left(Dx_{R}^{\left(l\right)}\right)V_{R}^{\left(l\right)}+U_{W}^{\left(l\right)}diag\left(Dx_{W}^{\left(l\right)}\right)V_{W}^{\left(l\right)}$$

(7) $$Y\approx \hat{Y}=U_{R}diag\left(Dy_{R}\right)H_{R}$$

where $\hat{X}$, $\hat{Y}$ are the estimated or reconstructed spectrograms of the input mixture and the respiratory training signal; ${\hat{X}}_{R}$, ${\hat{X}}_{W}$ are the estimated spectrograms of the RS and WS; $U_{R}$, $U_{W}$ are the estimated basis matrices of the RS and WS; $V_{R}$, $V_{W}$ are the estimated activation matrices of the RS and WS for the mixture; $H_{R}$ is the estimated activation matrix of the RS for the respiratory training signal. All of these matrices are non-negative matrices. The number of respiratory and wheezing components will be denoted as $K_{R}$ and $K_{W}$, respectively. The $L^{2}$-norm of each column of $U_{R}$ or $U_{W}$ is equal to 1.0. The terms $Dx_{R}$ and $Dx_{W}$ represent vectors with the $L^{2}$-norm of each activation component of RS and WS, respectively. Similarly, the term $Dy_{R}$ represents a vector with the $L^{2}$-norm of each activation component of RS. Therefore, the $L^{2}$-norm of each row of $V_{R}$, $V_{W}$ or $H_{R}$ be equal to 1.0 due to the normalization procedure at each iteration. The operator diag() is the diagonal matrix.

Figure 3 depicts those models with *L*-segments of the mixture spectrogram X and the respiratory training spectrogram Y. As mentioned in the key assumption (i), Equation (7) models the respiratory training reconstruction by letting the estimated basis matrix $U_{R}$ to contain spectral patterns that define the common behavior of RS. As mentioned in the key assumption (ii), Equation (6) aims to learn the common basis vectors $U_{R}$ of *L*-segments $X^{\left(1\right)}$,$X^{\left(2\right)}$, …, $X^{\left(L\right)}$ to model repetitive spectral components throughout the segments, since RS can be considered as repetitive sound events in human breathing. On the other hand, $U_{W}^{\left(l\right)}$ is responsible for recovering WS that can be contained in each segment. Combining the two previous factorization models, $U_{R}$ can model both spectral characteristics of the respiratory training Y and temporally repeating components belonging to the segments $X^{\left(1\right)}$,$X^{\left(2\right)}$, …,$X^{\left(L\right)}$. Considering the previous assumption (iii), the main contribution of the proposed method is to give greater importance, by means of weighting, to those segments classified as non-wheezing ($C^{\left(l\right)}=0$) in the NMPCF decomposition to learn more accurate the common basis vectors $U_{R}$ since these segments will not be interfered by WS so, the spectral modelling of RS will be more acoustically reliable. In Figure 3, the segments $X^{\left(2\right)}$ and $X^{\left(L\right)}$ are classified as non-wheezing segments.

The objective function of the proposed method IIS-NMPCF can be constructed to minimize the residuals of the models (6) and (7),

(8) $$\begin{array}{cc} \Gamma_{IIS-NMPCF}=\; & \overset{\mathrm{Objective}\mathrm{function}\mathrm{applied}\mathrm{to}\mathrm{the}\mathrm{set}\mathrm{of}L-\mathrm{segments}\mathrm{of}\mathrm{the}\mathrm{input}\mathrm{mixture}}{\overset{︷}{\sum_{l=1}^{L}\left[\lambda^{C^{\left(l\right)}}D_{KL}\left(X^{\left(l\right)}|{\hat{X}}^{\left(l\right)}\right)\right]+LD_{F}\left(U_{R}|0\right)+\sum_{l=1}^{L}\left[D_{F}\left(U_{W}^{\left(l\right)}|0\right)\right]}}\;\;\;\;\;\;+\\ & +\;\;\;\;\;\;\;\;\;\;\;\;\;\;\;\;\;\;\overset{\mathrm{Objective}\mathrm{function}\mathrm{applied}\mathrm{to}\mathrm{the}\mathrm{respiratory}\mathrm{training}}{\overset{︷}{\alpha D_{KL}\left(Y|\hat{Y}\right)+D_{F}\left(U_{R}|0\right)}} \end{array}$$

where $D_{KL}$ is the Kullback–Leibler divergence used to calculate the signal reconstruction error for each segment $D_{KL}\left(X^{\left(l\right)}|{\hat{X}}^{\left(l\right)}\right)$ and the respiratory training spectrogram $D_{KL}\left(Y|\hat{Y}\right)$. The penalization term $D_{F}$ represents the Frobenius norm applied to each basis matrix in order to prevent basis vectors from convergence to too small values [50]. The weighting factor $\lambda^{C^{\left(l\right)}}$ controls the relative importance of each segment matrix $X^{\left(l\right)}$ depending on the type of segment, wheezing ($C^{\left(l\right)}=1$) or non-wheezing ($C^{\left(l\right)}=0$), in the factorization model. The weighting factor α controls the relative importance of the respiratory training matrix Y in the factorization model.

Highlight that the weighting factor $\lambda^{C^{\left(l\right)}}$ plays a crucial role in the proposed method. The reason is because $\lambda^{C^{\left(l\right)}}$ controls the importance of which segments are more relevant in the modelling of the spectral patterns related to RS, specifically, those segments in which WS are not detected. Therefore, the following considerations about the parameter $\lambda^{C^{\left(l\right)}}$ must be taken into account:

(a) According to the estimated basis matrix $U_{R}$ or $U_{W}^{\left(l\right)}$, the weighting factor $\lambda^{C^{\left(l\right)}}$ can be classified as $\lambda_{R}^{C^{\left(l\right)}}$ or $\lambda_{W}^{C^{\left(l\right)}}$, respectively. As mentioned above, WS are always overlapped with RS so, we assume that none of the segments will model the behaviour of WS better than another. However, RS can be found isolated in some segments of human breathing due to the unpredictable nature of the pulmonary disorder. In this case, those segments in which WS are not contained will be more relevant to model the behaviour of RS. In this manner, $\lambda_{W}^{C^{\left(l\right)}}$ will set the same value for all segments, that is, $\lambda_{W}^{C^{\left(l\right)}}=\lambda_{W}$, l=1,…,L and $\lambda_{R}^{C^{\left(l\right)}}$ will be variable depending on the type of segment, wheezing ($C^{\left(l\right)}=1$) or non-wheezing ($C^{\left(l\right)}=0$), is analyzed. In addition, the value assigned to the weighing factors must satisfy $\lambda_{R}^{C^{\left(l\right)}}$ > $\lambda_{W}$ (see Section 4.4) since RS are always present in all segments of the input mixture and WS may not be.
(b) Focusing on the type of segment indicated by the parameter $C^{\left(l\right)}$, the weighting factor $\lambda_{R}^{C^{\left(l\right)}}$ can be classified as $\lambda_{R}^{0}$ or $\lambda_{R}^{1}$. The parameter $\lambda_{R}^{0}$ is associated with the non-wheezing segments ($C^{\left(l\right)}=0$) and $\lambda_{R}^{1}$ is associated with the wheezing segments ($C^{\left(l\right)}=1$). This allows to give greater importance to non-wheezing segments for the modeling of respiratory basis $U_{R}$. As consequence, the value assigned to the weighing factors must satisfy $\lambda_{R}^{0}$ > $\lambda_{R}^{1}$ (see Section 4.4).

Given the above, the estimated basis matrices $U_{R}$, $U_{W}^{\left(l\right)}$ and activations matrices $V_{R}^{\left(l\right)}$, $V_{W}^{\left(l\right)}$, $H_{R}$ can be obtained by applying a gradient descent algorithm based on multiplicative update rules as follows,

(9) $$U_{R}\leftarrow U_{R}⊙\frac{\sum_{l=1}^{L}\left[\lambda_{R}^{C^{\left(l\right)}}\left(X^{\left(l\right)}⊘{\hat{X}}^{\left(l\right)}\right){\left(diag\left(Dx_{R}^{\left(l\right)}\right)V_{R}^{\left(l\right)}\right)}^{T}\right]+\alpha \left(Y⊘\hat{Y}\right){\left(diag\left(Dy_{R}\right)H_{R}\right)}^{T}}{\sum_{l=1}^{L}\left[\lambda_{R}^{C^{\left(l\right)}}1_{F,T}{\left(diag\left(Dx_{R}^{\left(l\right)}\right)V_{R}^{\left(l\right)}\right)}^{T}\right]+\alpha 1_{F,T}{\left(diag\left(Dy_{R}\right)H_{R}\right)}^{T}+2\left(L+1\right)U_{R}}$$

(10) $$U_{W}^{\left(l\right)}\leftarrow U_{W}^{\left(l\right)}⊙\frac{\lambda_{W}\left(X^{\left(l\right)}⊘{\hat{X}}^{\left(l\right)}\right){\left(diag\left(Dx_{W}^{\left(l\right)}\right)V_{W}^{\left(l\right)}\right)}^{T}}{\lambda_{W}1_{F,T}{\left(diag\left(Dx_{W}^{\left(l\right)}\right)V_{W}^{\left(l\right)}\right)}^{T}+2U_{W}^{\left(l\right)}}$$

(11) $$V_{R}^{\left(l\right)}\leftarrow V_{R}^{\left(l\right)}⊙\frac{{\left(U_{R}diag\left(Dx_{R}^{\left(l\right)}\right)\right)}^{T}\left(X^{\left(l\right)}⊘{\hat{X}}^{\left(l\right)}\right)}{{\left(U_{R}diag\left(Dx_{R}^{\left(l\right)}\right)\right)}^{T}1_{F,T}}$$

(12) $$V_{W}^{\left(l\right)}\leftarrow V_{W}^{\left(l\right)}⊙\frac{{\left(U_{W}^{\left(l\right)}diag\left(Dx_{W}^{\left(l\right)}\right)\right)}^{T}\left(X^{\left(l\right)}⊘{\hat{X}}^{\left(l\right)}\right)}{{\left(U_{W}^{\left(l\right)}diag\left(Dx_{W}^{\left(l\right)}\right)\right)}^{T}1_{F,T}}$$

(13) $$H_{R}\leftarrow H_{R}⊙\frac{{\left(U_{R}diag\left(Dy_{R}\right)\right)}^{T}\left(Y⊘\hat{Y}\right)}{{\left(U_{R}diag\left(Dy_{R}\right)\right)}^{T}1_{F,T}}$$

The set of matrices $U_{R}$, $U_{W}^{\left(l\right)}$, $V_{R}^{\left(l\right)}$, $V_{W}^{\left(l\right)}$, $H_{R}$ are obtained updating the rules (9)–(13) until the algorithm converges or reaches a maximum number of iterations *M*. At each iteration, the activation matrices $V_{R}^{\left(l\right)}$, $V_{W}^{\left(l\right)}$, $H_{R}$ and the basis matrices $U_{R}$, $U_{W}^{\left(l\right)}$ must be normalized applying the $L^{2}$-norm (see Equation (14)). As a result, $Dx_{R}$, $Dx_{W}$, $Dy_{R}$ must be updated multiplying by the $L^{2}$-norm obtained at each previous normalization (see Equation (15)). The normalization process ensures that both the sum of the square elements of each k-th column of the basis matrices $U_{R}$, $U_{W}^{\left(l\right)}$ and the sum of the square elements of each k-th row of the activation matrices $V_{R}^{\left(l\right)}$, $V_{W}^{\left(l\right)}$, $H_{R}$ equals 1.0 [46].

(14) $$G\left(k\right)=\frac{G(k)}{\sqrt{\sum G^{2}\left(k\right)}}$$

(15) $$D_{J}\left(k\right)=D_{J}\left(k\right)\sqrt{\sum G^{2}\left(k\right)}$$

where (G, *J*, *k*) = {($U_{R}$, *R*, $k_{R}$), ($U_{W}^{\left(l\right)}$, *W*, $k_{W}$), ($V_{R}^{\left(l\right)}$, *R*, $k_{R}$), ($V_{W}^{\left(l\right)}$, *W*, $k_{W}$), ($H_{R}$, *R*, $k_{R}$)} respectively. If we consider the basis matrix $G=\left(U_{R},U_{W}^{\left(l\right)}\right)\to \sqrt{\sum G^{2}\left(k\right)}=\sqrt{\sum_{f=1}^{F}G^{2}\left(f,k\right)}$. If we consider the activation matrix $G=\left(V_{R}^{\left(l\right)},V_{W}^{\left(l\right)},H_{R}\right)\to \sqrt{\sum G^{2}\left(k\right)}=\sqrt{\sum_{t=1}^{T}G^{2}\left(k,t\right)}$.

After the updating process, the estimated spectrograms ${\hat{X}}_{R}^{\left(l\right)}$ and ${\hat{X}}_{W}^{\left(l\right)}$ for each segment can be reconstructed as follows:

(16) $${\hat{X}}_{R}^{\left(l\right)}=U_{R}diag\left(Dx_{R}^{\left(l\right)}\right)V_{R}^{\left(l\right)}$$

(17) $${\hat{X}}_{W}^{\left(l\right)}=U_{W}^{\left(l\right)}diag\left(Dx_{W}^{\left(l\right)}\right)V_{W}^{\left(l\right)}$$

Note that ${\hat{X}}_{R}^{\left(l\right)}$ and ${\hat{X}}_{W}^{\left(l\right)}$ must be denormalized by multiplying by the denominator of Equation (5). A Wiener filtering [32,55] has been applied in order to ensure a conservative signal reconstruction and to obtain the estimated complex wheezing and respiratory spectrogram of each segment. ${\hat{X}}_{R}$ and ${\hat{X}}_{W}$ are obtained by concatenating the estimated complex spectrograms of each segment, ${\hat{X}}_{R}\;=\;\left[{\hat{X}}_{R}^{\left(1\right)},{\hat{X}}_{R}^{\left(2\right)},\ldots ,{\hat{X}}_{R}^{\left(L\right)}\right]$ and ${\hat{X}}_{W}=\left[{\hat{X}}_{W}^{\left(1\right)},{\hat{X}}_{W}^{\left(2\right)},\ldots ,{\hat{X}}_{W}^{\left(L\right)}\right]$, respectively. Finally, the inverse overlap-add STFT is applied to synthesize the estimated RS signal $\hat{r}\left[n\right]$ and the estimated WS signal $\hat{w}\left[n\right]$ in time domain using the phase of the input mixture. The wheezing/normal respiratory sound separation procedure is summarized in Algorithm 1.
**Algorithm 1** Wheezing sound separation using IIS-NMPCF.**Require**: x[n], y[n], $K_{R}$, $K_{W}$, $\lambda_{R}^{0}$, $\lambda_{R}^{1}$, $\lambda_{W}$, α and *M*.1)Compute the normalized magnitude spectrogram X of the mixture x[n].2)Compute the normalized magnitude spectrogram Y of the training y[n].3)Divide the spectrogram X into *L*-segments $X^{\left(1\right)}$, $X^{\left(2\right)}$, …, $X^{\left(L\right)}$ using AMIE_SEG [53].4)Classify the *L*-segments into wheezing ($C^{\left(l\right)}=1$) and non-wheezing ($C^{\left(l\right)}=0$) using a wheezing detection algorithm [54].5)Initialize each activation and basis matrix $U_{R}$, $U_{W}^{\left(l\right)}$, $V_{R}^{\left(l\right)}$, $V_{W}^{\left(l\right)}$, $H_{R}$ with random non-negative values.6)Update each activation and basis matrix $U_{R}$, $U_{W}^{\left(l\right)}$, $V_{R}^{\left(l\right)}$, $V_{W}^{\left(l\right)}$, $H_{R}$ using Equations (9)–(13) for the predefined number of iterations *M*. At each iteration, normalize each activation and basis matrix $U_{R}$, $U_{W}^{\left(l\right)}$, $V_{R}^{\left(l\right)}$, $V_{W}^{\left(l\right)}$, $H_{R}$ and update the terms $Dx_{R}$, $Dx_{W}$ and $Dy_{R}$ using Equations (14) and (15).7)Compute the estimated magnitude spectrograms ${\hat{X}}_{R}^{\left(l\right)}$ and ${\hat{X}}_{W}^{\left(l\right)}$.8)Denormalize the estimated magnitude spectrograms ${\hat{X}}_{R}^{\left(l\right)}$ and ${\hat{X}}_{W}^{\left(l\right)}$.9)Apply a Wiener filtering [32] on ${\hat{X}}_{R}^{\left(l\right)}$ and ${\hat{X}}_{W}^{\left(l\right)}$.10)Concatenate all the estimated complex respiratory spectrograms: ${\hat{X}}_{R}=\left[{\hat{X}}_{R}^{\left(1\right)},{\hat{X}}_{R}^{\left(2\right)},\ldots ,{\hat{X}}_{R}^{\left(L\right)}\right]$.11)Concatenate all the estimated complex wheezing spectrograms: ${\hat{X}}_{W}=\left[{\hat{X}}_{W}^{\left(1\right)},{\hat{X}}_{W}^{\left(2\right)},\ldots ,{\hat{X}}_{W}^{\left(L\right)}\right]$.12)Synthesize $\hat{r}\left[n\right]$.13)Synthesize $\hat{w}\left[n\right]$.          **return** $\hat{r}\left[n\right]$ and $\hat{w}\left[n\right]$

## 4. Experimental Results

### 4.1. Dataset and Metric

Because there is no public database where only wheeze sounds can be found to the best of our knowledge, two datasets P1 and T1 (T1H, T1M and T1L), detailed in Table 1, have been used in the evaluation of the proposed method with a total of 64 recordings considering the two databases. Specifically, the database P1 consists of 48 recordings (that is, 3/4 of the total recordings used in the experiments) and the database T1 consists of 16 recordings (that is, 1/4 of the total recordings used in the experiments). The dataset P1 has been used in the hyperparametric optimization process (see Section 4.4) while the dataset T1 has been used in the separation testing (see Section 4.5). The databases P1 and T1 have been created by collecting a set of recordings from different subjects of the most widely used Internet pulmonary repositories [56,57,58,59,60,61,62,63,64,65,66,67,68]. These recordings, captured from the trachea, anterior, and posterior chest using either a stethoscope or microphone, were collected from subjects with different pathologies, including asthma, bronchitis or COPD. The databases P1 and T1 have been created by randomly selecting recordings from the above-mentioned repositories. It must be highlighted that P1 is not a part of T1 in order to validate the results. Therefore, the recordings selected for the database P1 are not the same as the recordings selected for the database T1. In total, these databases provide 1474 s of recording, 96 unhealthy subjects, 874 respiratory events (a respiratory event is defined as inspiration or expiration) and 133 wheezes. Note that each recording has been created using single-channel configuration, a sampling rate equals 2048 Hz and a bit resolution of 16 bits.

Specifically, the datasets P1 and T1 (T1H, T1M and T1L) have been created mixing only WS recordings manually separated w[n], in which respiratory sounds are inactive, and only RS recordings r[n], in which wheezing sounds are inactive, obtained from the above-mentioned repositories. Highlight that wheezing sounds cannot be recorded isolated since WS are always overlapped with RS, that is, both sounds are produced by the same bronchial tree in the lungs. To do this, a MATLAB tool, designed by the authors, has been used to visually modify the spectrogram values. Specifically, this tool behaves as an eraser that allows us, by means of the mouse, to set to zero those bins of the spectrogram that we observe that do not belong to a wheeze sound, a fact that is also verified by a listening inspection of the resulting signal. Therefore, only the bins corresponding to WS have been kept active for each signal w[n]. Both the fundamental component of WS and its corresponding harmonics have been considered. Note that the recordings used to create the database P1 are different from those used to create the database T1.

The datasets T1H (SNR = 5 dB), T1M (SNR = 0 dB) and T1L (SNR = −5 dB) are composed of the same set of signals w[n] and r[n] but they have been mixed using a different Signal-to-Noise Ratio (SNR). Specifically, T1H is composed of mixtures in which the power of w[n] is 5 dB greater compared to r[n] so, WS are louder than RS. The dataset T1M is composed of mixtures in which the power of both w[n] and r[n] is the same so, both type of sounds is similarly audible. Finally, the dataset T1L is composed of mixtures in which the power of w[n] is 5 dB lower compared to r[n] so, RS are louder than WS. Note that in each mixture process, the power related to w[n] and r[n] are calculated and the signal with the highest power is left fixed while the signal with the lowest power is scaled to obtain the desired SNR in order to avoid audio saturation or distortion in the signal scaling process.

To assess the sound separation performance of the proposed method, the BSS EVAL toolbox [69,70] has been applied because it is widely used in the field of sound source separation. The metrics used are the following: (1) Source-to-distortion ratio (SDR), which provides information on the overall quality of the separation process; (2) Source-to-interferences ratio (SIR), which reports the presence of WS contained in RS and vice versa; and (3) Source-to-artifacts ratio (SAR), which provides information on the artifacts in the separated signal from separation and/or resynthesis. The principle to obtain the value of these metrics is to decompose the total error, between the estimated target signal $\hat{s}\left[n\right]$ and the original target signal s[n], in three terms related to three types of error, as follows [70]:

(18) $$\hat{s}\left[n\right]-s\left[n\right]={e_{s}}^{interf}\left[n\right]+{e_{s}}^{artifacts}\left[n\right]+{e_{s}}^{spatial}\left[n\right]$$

where ${e_{s}}^{interf}\left[n\right]$ is the error term related to the interference produced by the unwanted sources; ${e_{s}}^{artifacts}\left[n\right]$ is the error term attributed to the artifacts generated by the separation algorithm; and ${e_{s}}^{spatial}\left[n\right]$ is the error term attributed to spatial distortion. We can now define the SDR, SIR and SAR values, expressed in dB, as follows:

(19) $$SDR=10\log_{10}\frac{{∥s[n]∥}^{2}}{{∥{e_{s}}^{interf}\left[n\right]+{e_{s}}^{artifacts}\left[n\right]+{e_{s}}^{spatial}\left[n\right]∥}^{2}}$$

(20) $$SIR=10\log_{10}\frac{{∥s[n]∥}^{2}}{{∥{e_{s}}^{interf}\left[n\right]∥}^{2}}$$

(21) $$SAR=10\log_{10}\frac{{∥s\left[n\right]+{e_{s}}^{interf}\left[n\right]+{e_{s}}^{spatial}\left[n\right]∥}^{2}}{{∥{e_{s}}^{artifacts}\left[n\right]∥}^{2}}$$

Note that the term *s* indicates the target signal to be analyzed. In this article *s* could be the wheezing signals (s=w) and the respiratory signals (s=r). Therefore, in the case of the wheezing signals ($\hat{s}\left[n\right]$, s[n], ${e_{s}}^{interf}\left[n\right]$, ${e_{s}}^{artifacts}\left[n\right]$, ${e_{s}}^{spatial}\left[n\right]$) = ($\hat{w}\left[n\right]$, w[n], ${e_{w}}^{interf}\left[n\right]$, ${e_{w}}^{artifacts}\left[n\right]$, ${e_{w}}^{spatial}\left[n\right]$) and in the case of the respiratory signals ($\hat{s}\left[n\right]$, s[n], ${e_{s}}^{interf}\left[n\right]$, ${e_{s}}^{artifacts}\left[n\right]$, ${e_{s}}^{spatial}\left[n\right]$) = ($\hat{r}\left[n\right]$, r[n], ${e_{r}}^{interf}\left[n\right]$, ${e_{r}}^{artifacts}\left[n\right]$, ${e_{r}}^{spatial}\left[n\right]$). The estimated signals $\hat{w}\left[n\right]$, $\hat{r}\left[n\right]$ are obtained by the separation algorithm, the original signals w[n], r[n] are obtained from the original separated signals used in the creation of the mixtures of the databases and the error terms are obtained using the BSS EVAL toolbox. We refer the reader to [70] for more details.

In this article, three different sets of SDR, SIR and SAR metrics will be analyzed as follows: (i) SDR${}_{w}$, SIR${}_{w}$ and SAR${}_{w}$ are referred to WS, (ii) SDR${}_{r}$, SIR${}_{r}$ and SAR${}_{r}$ are referred to RS; and (iii) SDR${}_{m}$ is associated to the average considering SDR${}_{w}$ and SDR${}_{r}$, SIR${}_{m}$ is associated to the average considering SIR${}_{w}$ and SIR${}_{r}$, and SAR${}_{m}$ is associated to the average considering SAR${}_{w}$ and SAR${}_{r}$.

### 4.2. Experiments Setup

According to the results obtained in similar works [32,54] related to wheezing sound analysis, the following parameters provided the best trade-off between the separation performance and the computational cost: sampling rate $f_{s}=2048$ Hz, Hamming window with N=256 samples length and 25% overlap (temporal resolution of 31.3 ms), and a discrete Fourier transform using 2N points.

The performance of the proposed method depends on the initial values with which each activation and basis matrix is initialized. For this reason, we have evaluated four times each input mixture with the proposed method and therefore, the results are averaged values. Furthermore, the convergence of the proposed method was empirically achieved after 50 iterations for all mixtures, so M = 50 iterations.

### 4.3. Comparison Methods

A set of reference baseline sound source separation methods have been compared to assess the sound separation performance achieved by the proposed method (IIS-NMPCF). As mentioned in Section 2, these methods can be divided into two groups: (i) NMF-based methods (NMF, SNMF, SSNMF and CNMF); and (ii) NMPCF-based methods (1S-NMPCF, 2S-NMPCF, T-NMPCF and ST-NMPCF). Highlight that the main parameters of the previous baseline methods have been optimized using the database P1. However, the following considerations must be taken into account to a fair comparison:

- A training signal y[n], created to simulate the behavior of RS, is used in the baseline methods SNMF, SSNMF, 1S-NMPCF, 2S-NMPCF, ST-NMPCF and the proposed method IIS-NMPCF. The training signal y[n] has been created by concatenating randomly a set of normal respiratory stages only composed of RS obtained from the previously mentioned Internet pulmonary repositories [56,57,58,59,60,61,62,63,64,65,66,67,68]. Specifically, the signal y[n] has a temporal duration of 128 s and 54 respiratory stages (inspiration or expiration). Note that the normal respiratory stages used to construct y[n] do not correspond to any of the respiratory stages used in the databases P1 or T1.
- SNMF and 2S-NMPCF must use a training signal to simulate the behaviour of wheezing sounds. Taking into account that WS can be defined as continuous adventitious sounds that show a pitched sound (see Section 1), a signal z[n] has been created by concatenating a set of single pitches located along the frequency band 100 Hz–1000 Hz in which WS are typically present. Each pitch is represented by a sinusoidal signal multiplied by a Hamming window of *N* samples. The distance between the frequencies of each pitch is equal to the value provided by the spectral spacing of the model. Considering that all evaluated methods have used the same parameters previously mentioned in Section 4.2, the spectral spacing equals to 4 Hz.
- T-NMPCF and ST-NMPCF as well as IIS-NMPCF has been implemented using AMIE_SEG [53] to divide the input spectrogram X into the *L*-segments $X^{\left(1\right)}$,$X^{\left(2\right)}$, …, $X^{\left(L\right)}$.
- CNMF has been evaluated using its optimal parameters found in [32].

### 4.4. Optimization

The proposed method employs a wide range of parameters $K_{R}$, $K_{W}$, α, $\lambda_{W}$, $\lambda_{R}^{0}$ and $\lambda_{R}^{1}$ that can affect significantly the separation performance and the reconstructed sound quality. A hyperparametric optimization procedure has been applied to the main parameters of the proposed method IIS-NMPCF to obtain the optimal parameters that maximize the audio quality of the estimated wheezing signal $\hat{w}\left[n\right]$. In this work, a preliminary evaluation using visual inspection reduced the parameter space as follows: $K_{R}$ = (8, 16, 32, 64, 128, 256, 512), $K_{W}$ = (8, 16, 32, 64, 128, 256, 512), α = (0, 0.01, 0.1, 1, 10, 100), $\lambda_{W}$ = (0.001, 0.01, 0.1, 1, 10), $\lambda_{R}^{0}$ = (0.001, 0.01, 0.1, 1, 10, 100) and $\lambda_{R}^{1}$ = (0.001, 0.01, 0.1, 1, 10, 100).

The hyperparametric procedure is performed for each mixture of the dataset P1 in order to obtain the audio quality of the estimated wheeze signal $\hat{w}\left[n\right]$ in terms of SDR${}_{w}$, SIR${}_{w}$ and SAR${}_{w}$. This procedure has been computed by evaluating all the possible combinations of the parameters $K_{R}$, $K_{W}$, α, $\lambda_{W}$, $\lambda_{R}^{0}$ and $\lambda_{R}^{1}$ that can be found within the parameter space defined above, providing the SDR${}_{w}$, SIR${}_{w}$ and SAR${}_{w}$ average values for each combination of parameters. Table 2 shows the optimal combination of the previous parameters that provides the best separation performance in terms of SDR${}_{w}$. Specifically, the optimal parameters corroborate our previous assumptions described in Section 3.2: (i) the highest weighting factor $\lambda_{R}^{0}=10$ is due to the high importance of the non-wheezing segments in the factorization of the respiratory bases since RS can be modeled by sharing spectral patterns that can be found in all non-wheezing segments during the breathing process; (ii) the second highest weighting factor α=1 is associated with the training signal since RS typically show common spectral behavior; (iii) the low weighting factor $\lambda_{R}^{1}=0.1$ is associated with the wheezing segments in the factorization of the respiratory bases since WS can interfere in the RS reconstruction; and (iv) the lowest weighting factor $\lambda_{W}=0.01$ is due to none of the *L*-segments is only composed by isolated WS.

Focusing on the parameter space defined above and keeping the optimal parameters shown in Table 2, the aim of the rest of the section is to analyze the stability and efficiency of the proposed method when its main parameters $K_{W}$, $K_{R}$, α, $\lambda_{W}$, $\lambda_{R}^{0}$ and $\lambda_{R}^{1}$ are distanced from the optimal values.

**Figure 4:**
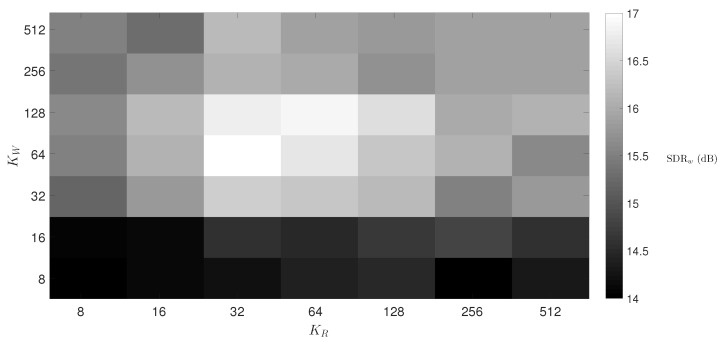
SDR${}_{w}$ average results from the hyperparametric optimization of the proposed method varying the parameters $K_{W}$ and $K_{R}$. The rest of parameters are the following: $\lambda_{R}^{0}=10$, α=1, $\lambda_{R}^{1}=0.1$ and $\lambda_{W}=0.01$.

Figure 4 shows the SDR${}_{w}$ results varying the number of respiratory $K_{R}$ and wheezing $K_{W}$ components. Figure 4 shows that the difference, in terms of SDR${}_{w}$, between the configuration of the parameters $K_{R}$ and $K_{W}$ that provides the best performance (SDR${}_{w}$ = 16.99 dB) and the worst performance (SDR${}_{w}$ = 14.01 dB) is approximately 3 dB. Therefore, the proposed method is stable within the defined parameter space $K_{W}$ and $K_{R}$ since the maximum loss that the algorithm can suffer is less than 3 dB regardless of the number of wheezing $K_{W}$ and respiratory $K_{R}$ components evaluated. Besides, the difference in SDR${}_{w}$ results is marginal (less than 0.2 dB) either using $K_{W}\geq 256$ and $K_{R}\geq 256$ or (less than 0.3 dB) using $K_{W}\leq 16$ and $K_{R}\leq 16$. Highlight that the proposed factorization model needs a minimum of respiratory and wheezing components so that WS and RS can be modelled correctly. An empirical analysis showed that the SDR${}_{w}$ results start to drop significantly when $K_{W}<16$ and $K_{R}<16$. Figure 4 shows that SDR${}_{w}$ results increase when the number of wheezing components is greater than the number of respiratory components ($K_{W}>K_{R}$). Specifically, comparing the parameter space $K_{W}\in \left[32-512\right]$ and $K_{R}\in \left[8-16\right]$ with $K_{W}\in \left[8-16\right]$ and $K_{R}\in \left[32-512\right]$, the performance of the method, in terms of SDR${}_{w}$, improves by about 1.7 dB. As a result, RS seem to be modelled with a lower number of bases than WS. Finally, the best performance of the proposed method IIS-NMPCF can be found in the parameter space comprised by $K_{W}\in \left[32-128\right]$ and $K_{R}\in \left[32-128\right]$ with SDR${}_{w}$ results above 16.5 dB. As previously indicated in Table 2, the proposed method provides its highest wheezing separation performance, SDR${}_{w}$ = 16.99 dB, using $K_{W}=64$ and $K_{R}=32$.

**Figure 5:**
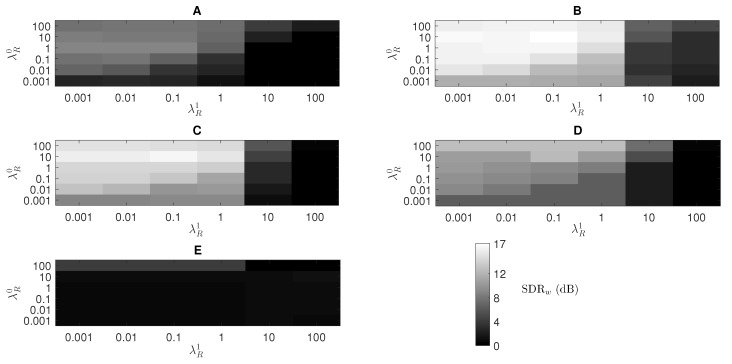
SDR${}_{w}$ average results from the hyperparametric optimization of the proposed method varying the parameters $\lambda_{W}$, $\lambda_{R}^{0}$ and $\lambda_{R}^{1}$. The rest of parameters are the following: $K_{W}=64$, $K_{R}=32$ and α=1. (**A**) $\lambda_{W}=0.001$, (**B**) $\lambda_{W}=0.01$, (**C**) $\lambda_{W}=0.1$, (**D**) $\lambda_{W}=1$ and (**E**) $\lambda_{W}=10$.

Figure 5 shows the optimization of the parameters $\lambda_{W}$, $\lambda_{R}^{0}$ and $\lambda_{R}^{1}$ of the proposed method in terms of SDR${}_{w}$ results, of the proposed method. Figure 5E shows a poor wheezing separation when the proposed method uses a $\lambda_{W}=10$ since the performance of the proposed method decreases exponentially (below 2 dB) in this scenario. The reason seems to indicate that WS are always overlapped with RS since both are produced by the same airflow through the bronchial tree of the lungs. Therefore, the proposed method wrongly models the wheeze bases when $\lambda_{W}\geq 10$ since it assumes that the *L*-segments of the input mixture are composed mostly of prominent WS. Figure 5A shows that SDR${}_{w}$ results decrease significantly when $\lambda_{W}=0.001$. In this case, the use of an excessively low weighting factor makes WS less important in the factorization process, causing that the separation process is not performed correctly since the estimated respiratory signal $\hat{r}\left[n\right]$ contains both WS and RS. Figure 5B,D show the lower and upper limit of the weighting factor $\lambda_{W}$ so that the performance of the method is not drastically affected. Figure 5 shows an improvement of the wheeze separation performance of the proposed method when $\lambda_{R}^{0}>\lambda_{R}^{1}$. Results suggest that, unlike the wheezing segments, the non-wheezing segments improve the modeling of the RS bases since these segment do not contain wheeze content so, they are not interfered by WS. As a result, $\lambda_{R}^{0}$ must be greater than $\lambda_{R}^{1}$ to increase the quality of the reconstructed respiratory signal $\hat{r}\left[n\right]$. In the parameter space comprised by $\lambda_{R}^{0}\in \left[0.001-100\right]$ and $\lambda_{R}^{1}\in \left[10-100\right]$, the SDR${}_{w}$ results are reduced significantly as can be seen in Figure 5. Therefore, a remarkable increase of $\lambda_{R}^{1}$ causes that the factorization model inserts a large proportion of wheezing interferences into the reconstructed respiratory signal. This fact produces more of the WS to be present in the reconstructed respiratory signal $\hat{r}\left[n\right]$ rather than in the reconstructed wheezing signal $\hat{w}\left[n\right]$. It can be observed that the maximum SDR${}_{w}$ value, approximately equal to 17 dB in Figure 5B, is provided by the proposed method for the set of parameters $\lambda_{W}=0.01$, $\lambda_{R}^{1}=0.1$ and $\lambda_{R}^{0}=10$. This optimization process confirms the assumptions introduced in Section 3.2. Firstly, the proposed method provides the greatest importance, with a weighting factor of $\lambda_{R}^{0}=10$, to the non-wheezing segments for the factorization of the basis matrix related to RS. Secondly, the proposed method provides less importance, with a weighing factor of $\lambda_{R}^{1}=0.1$, to the wheezing segments for the factorization of the basis matrix of the RS. Finally, the proposed method provides the least importance, with a weighting factor of $\lambda_{W}=0.01$, to the *L*-segments that composes the input mixture signal for the factorization of the basis matrix of WS, as in none of these segments are WS isolated.

Note that when $\lambda_{W}=\lambda_{R}^{0}=\lambda_{R}^{1}$, the proposed method works similarly to the conventional NMPCF approach, that is, ST-NMPCF. In particular, Figure 5B shows that ST-NMPCF obtains a SDR${}_{w}$ result equal to 13 dB (4 dB less than the optimal value obtained with the proposed method) using $\lambda_{W}=\lambda_{R}^{0}=\lambda_{R}^{1}=0.01$. This improvement provided by the proposed method confirms that adding different weighting factors to different segments of the input mixture into the NMPCF factorization enhances the acoustic fidelity of the spectral content of both RS and WS in the sound separation.

**Figure 6:**
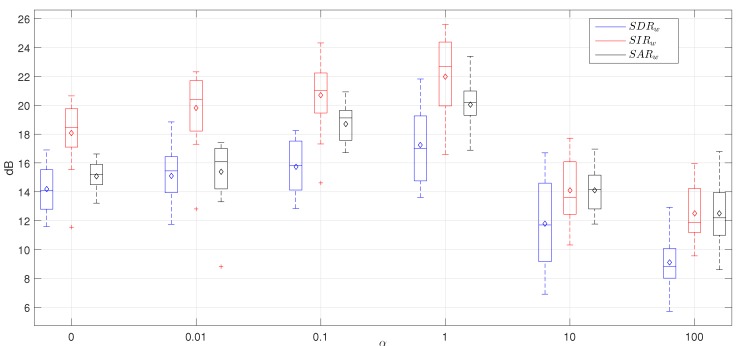
SDR${}_{w}$, SIR${}_{w}$ and SAR${}_{w}$ average results from the hyperparametric optimization of the proposed method varying the parameter α. The rest of parameters are the following: $K_{W}=64$, $K_{R}=32$, $\lambda_{R}^{0}=10$, $\lambda_{R}^{1}=0.1$ and $\lambda_{W}=0.01$.

Focusing on the importance of the respiratory training signal y[n] in the proposed IIS-NMPCF approach, Figure 6 shows SDR${}_{w}$, SIR${}_{w}$ and SAR${}_{w}$ results of the estimated wheezing signal evaluating the parameter space of the weighting factor α. Each box represents 48 data points, one for each mixture of the optimization dataset P1: each blue box represents the analysis for SDR${}_{w}$ values; each red box represents the analysis for SIR${}_{w}$ values; and each black box represents the analysis for SAR${}_{w}$ values. The lower and upper lines of each box show the 25th and 75th percentiles. The line in the middle of each box represents the median value. The diamond in the center of each box represents the average value. The lines extending above and below each box show the extent of the rest of the samples, excluding outliers. Outliers are defined as points that are over 1.5 times the interquartile range from the sample median, which are shown as crosses. The proposed method using α=0, henceforth called IIS${}_{0}$-NMPCF, does not use any training to model the respiratory bases. IIS${}_{0}$-NMPCF shows an efficient performance with an average separation results of SDR${}_{w}$ = 14 dB, SIR${}_{w}$ = 18 dB and SAR${}_{w}$ = 15 dB. Based on these results, it can be confirmed that IIS${}_{0}$-NMPCF maintains a remarkable performance in the quality of the estimated wheezing signal $\hat{w}\left[n\right]$. However, the best average separation results, SDR${}_{w}$ = 17 dB, SIR${}_{w}$ = 22 dB and SAR${}_{w}$ = 20 dB, are obtained using α=1. The optimal configuration of the proposed method IIS-NMPCF (α=1) produces a significant improvement of 3 dB in SDR${}_{w}$, 4 dB in SIR${}_{w}$ and 5 dB in SAR${}_{w}$ compared to IIS${}_{0}$-NMPCF. As a result, two conclusions are stated: (i) the performance of IIS-NMPCF is mainly due to the importance of the different segments depending on the presence or absence of WS so, not using any respiratory training signal the method maintains good separation results; and (ii) the use of a respiratory training signal significantly improves the performance of the proposed method IIS-NMPCF since it is combined both the information provided by the spectral patterns found at inter-segments with the information provided by the spectral patterns found in the respiratory training signal. This fact implies that the probability of finding wheezing interferences in the factorized respiratory bases decreases considerably.

Moreover, SDR${}_{w}$, SIR${}_{w}$ and SAR${}_{w}$ results, obtained using α>10, suffer a significant decrease compared to the best performance provided by the proposed method (α=1) as shown in Figure 6. In this case ($\alpha >\lambda_{R}^{0}$), the factorization gives more importance to the spectral patterns obtained from the respiratory training signal instead of the spectral patterns shared between the different segments, that is, the proposed method IIS-NMPCF performs similarly to 1S-NMPCF.

### 4.5. Results and Discussion

This section assesses the sound quality of the estimated or reconstructed WS and RS obtained by the proposed method (IIS${}_{0}$-NMPCF and IIS-NMPCF) and the baseline separation NMF-based and NMPCF-based methods described in Section 2. Table 3 describes the methods evaluated, indicating the approach on which they are based and the spectro-temporal information used in the modelling of WS and RS.

**Figure 7:**
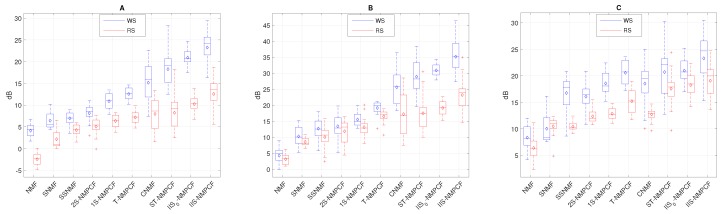
SDR${}_{w}$ and SDR${}_{r}$ results (**A**), SIR${}_{w}$ and SIR${}_{r}$ results (**B**) and SAR${}_{w}$ and SAR${}_{r}$ results (**C**) evaluating the dataset T1H (SNR = 5 dB). Note that SDR${}_{w}$, SIR${}_{w}$ and SAR${}_{w}$ are represented by blue boxes while SDR${}_{r}$, SIR${}_{r}$ and SAR${}_{r}$ are represented by red boxes.

**Figure 8:**
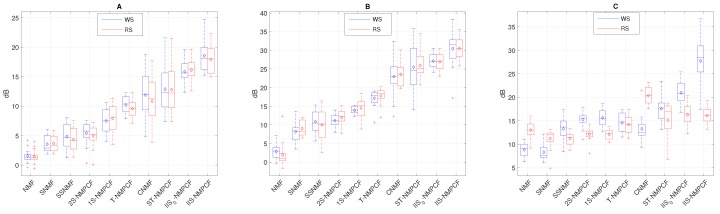
SDR${}_{w}$ and SDR${}_{r}$ results (**A**), SIR${}_{w}$ and SIR${}_{r}$ results (**B**) and SAR${}_{w}$ and SAR${}_{r}$ results (**C**) evaluating the dataset T1M (SNR = 0 dB). Note that SDR${}_{w}$, SIR${}_{w}$ and SAR${}_{w}$ are represented by blue boxes while SDR${}_{r}$, SIR${}_{r}$ and SAR${}_{r}$ are represented by red boxes.

**Figure 9:**
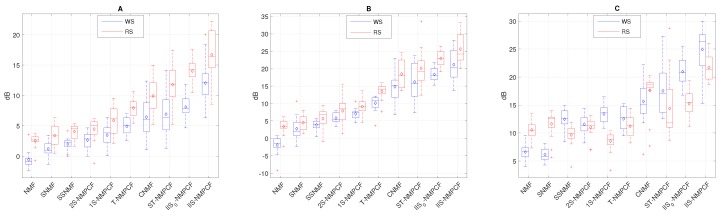
SDR${}_{w}$ and SDR${}_{r}$ results (**A**), SIR${}_{w}$ and SIR${}_{r}$ results (**B**) and SAR${}_{w}$ and SAR${}_{r}$ results (**C**) evaluating the dataset T1L (SNR = −5 dB). Note that SDR${}_{w}$, SIR${}_{w}$ and SAR${}_{w}$ are represented by blue boxes while SDR${}_{r}$, SIR${}_{r}$ and SAR${}_{r}$ are represented by red boxes.

Next, SDR, SIR and SAR results of the estimated wheezing signal $\hat{w}\left[n\right]$ and the estimated respiratory signal $\hat{r}\left[n\right]$ obtained by the proposed method and the aforementioned baseline methods evaluating the testing datasets T1H (see Figure 7), T1M (see Figure 8) and T1L (see Figure 9) are analyzed to extract interesting information about the sound separation performance of the methods evaluated. Each blue box corresponds to the SDR${}_{w}$, SIR${}_{w}$ and SAR${}_{w}$ results of the estimated wheezing signal while each red box corresponds to the SDR${}_{r}$, SIR${}_{r}$ and SAR${}_{r}$ results of the estimated respiratory signal. Note that the methods have been shown sorted from lowest to highest separation performance to represent results as a ranking. The following information can be derived from the analysis of results from Figure 7, Figure 8 and Figure 9:

- The decrease in SNR affects significantly the SDR and SIR results for both WS and RS. Focusing on Figure 7 in which SNR = 5 dB, results tend to be higher for reconstructed WS compared to the reconstructed RS because WS are louder than RS, so the sound separation benefits the audio quality of the reconstructed WS. Focusing on Figure 8 in which SNR = 0 dB, results for both WS and RS tend to remain stable because both WS and RS are similarly audible, so the performance of the sound separation seems to work equally between WS and RS. However, in Figure 9 in which SNR = −5 dB, results tend to be better for reconstructed RS since RS are louder than WS. This decrease in SNR implies that SDR${}_{m}$ and SIR${}_{m}$ results are worse in T1L compared to T1H. The reason is because RS are louder than WS when SNR < 0 dB (T1L) and as a consequence, WS be inaudible in this acoustic scenario so, the reduction of the SNR implies a greater time-frequency overlapping from RS to WS than the opposite.
- The standard NMF is ranked at the bottom, obtaining the worst sound separation performance since it achieves the signal reconstruction but not a factorization composed of audio events with physical meaning. The standard NMF cannot group the factorized bases to the sound source that generated them unlike the other methods because the standard NMF does not incorporate any type of information into the factorization process to model the spectro-temporal characteristics shown by WS and RS.
- Semi-supervised approaches (SSNMF and 1S-NMPCF) obtain better performance compared to supervised approaches (SNMF and 2S-NMPCF). Regardless of the approach, NMF or NMPCF, the use of the RS training signal is more effective that the use of both RS and WS training signals. It indicates that both training signals provide over-information that causes spectro-temporal ambiguity in the factorization of both WS and RS dictionaries.
- NMPCF-based methods (1S-NMPCF) obtain better separation performance than NMF-based methods (SSNMF). This fact seems to be because SSNMF uses a fixed dictionary composed of respiratory bases previously trained. However, 1S-NMPCF does not need a previous training stage, since it applies a joint matrix factorization using the input mixture and the respiratory training to obtain a dynamic dictionary of respiratory bases shared between both signals, obtaining a different dictionary of bases for each input mixture.
- Comparing NMPCF-based methods, T-NMPCF improves the separation performance compared to 1S-NMPCF. Results suggest that the dictionary of respiratory bases is more efficient when the input mixture is divided into segments in order to find repetitive patterns of RS.
- ST-NMPCF, the combination of the approaches 1S-NMPCF and T-NMPCF, obtains a significant improvement of the wheezing separation performance. Specifically, SDR${}_{w}$ = 5.96 dB and SIR${}_{w}$ = 9.73 dB evaluating T1H (Figure 7). It indicates that a more reliable modelling of RS can be achieved using jointly the shared respiratory spectral patterns along the segments and a prior knowledge of the respiratory spectral content by means of the respiratory training signal.
- CNMF [32] obtains competitive SDR SIR and SAR results compared to the methods above, ranking fourth. In some cases, WS and RS are modelled efficiently by applying its proposed constraints, but in other cases in which WS and RS are uncommon, CNMF does not model properly the spectro-temporal behavior of the target sounds.

Focusing on the main contribution proposed in this work, the incorporation of higher importance to those segments classified as non-wheezing in the co-factorization process, Figure 7, Figure 8 and Figure 9 reveal the following information:

- A significant separation performance improvement over the conventional T-NMPCF and ST-NMPCF is achieved adding greater importance to the non-wheezing segments in the co-factorization process. The SDR${}_{w}$ improvement of IIS${}_{0}$-NMPCF over T-NMPCF is about 8.31 dB (T1H), 5.18 dB (T1M) and 4.85 dB (T1L). The SIR${}_{w}$ improvement of IIS${}_{0}$-NMPCF over T-NMPCF is about 11.09 dB (T1H), 10.18 dB (T1M) and 8.33 dB (T1L). The SDR${}_{w}$ improvement of IIS${}_{0}$-NMPCF over ST-NMPCF is about 2.67 dB (T1H), 3.03 dB (T1M) and 1.69 dB (T1L). The SIR${}_{w}$ improvement of IIS${}_{0}$-NMPCF over ST-NMPCF is about 1.98 dB (T1H), 2.25 dB (T1M) and 1.87 dB (T1L). Results suggest that the inclusion of inter-segment information into the co-factorization process for modeling repetitive RS improves significantly the separation performance because it avoids that the respiratory spectral patterns obtained from the factorization remaining uncontaminated in wheezing segments.
- Adding prior knowledge of RS to IIS${}_{0}$-NMPCF improves significantly the sound separation performance. The SDR${}_{w}$ improvement of IIS-NMPCF over IIS${}_{0}$-NMPCF is about 3.07 dB (T1H), 2.89 dB (T1M) and 4.12 dB (T1L). The SIR${}_{w}$ improvement of IIS-NMPCF over IIS${}_{0}$-NMPCF is about 4.96 dB (T1H), 3.23 dB (T1M) and 3.02 dB (T1L). However, the dispersion between SDR and SIR results increases when the respiratory training signal is incorporated into the co-factorization process.

Focusing on the SAR results observed in Figure 7C, Figure 8C and Figure 9C: (i) NMPCF-based methods produce fewer artifacts than NMF-based methods; (ii) the spectro-temporal information used in the modelling of WS and RS allows to reduce the ambiguity that NMPCF-based methods are affected by decreasing the amount of artifacts. For this reason, the proposed method IIS-NMPCF, which uses more spectro-temporal information to model RS compared to the other NMPCF-based methods, obtains the best separation performance in terms of SAR.

In order to guarantee the relevance of the respiratory and wheezing SDR, SIR and SAR results shown in Figure 7, Figure 8 and Figure 9, an analysis of the statistical significance, using an one-side paired *t*-test, has been performed comparing the proposed method (IIS-NMPCF) with the rest of the evaluated methods as shown in Table 4, Table 5 and Table 6. It can be observed that results confirm the significant improvement obtained by IIS-NMPCF compared to the other evaluated methods.

**Figure 10:**
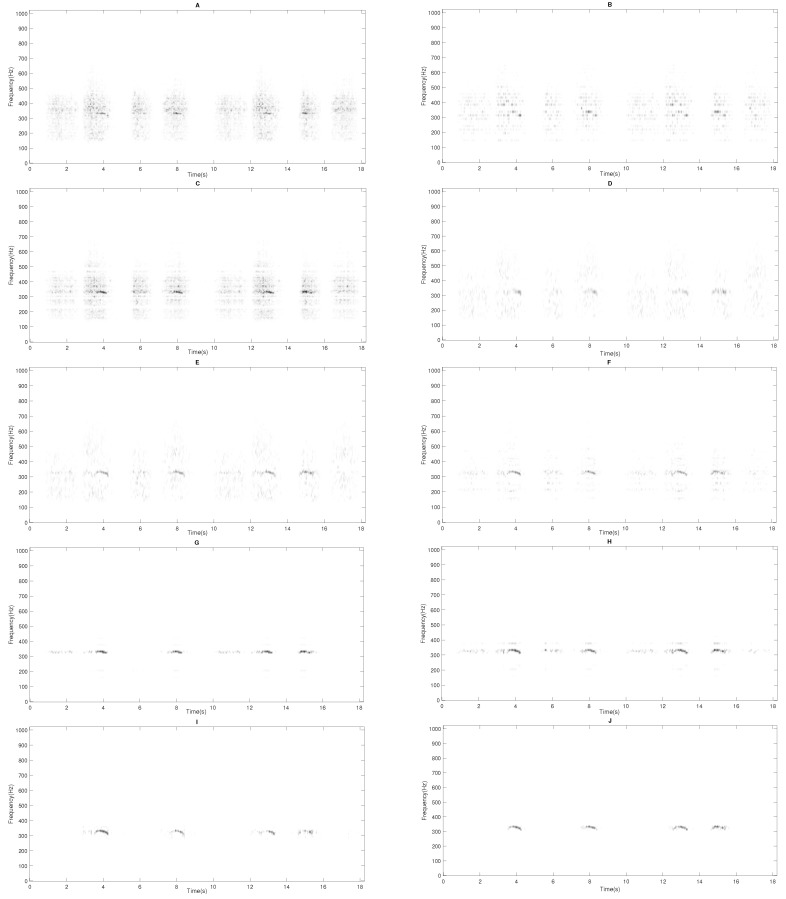
The estimated wheezing spectrogram ${\hat{X}}_{W}$ obtained from the input spectrogram X shown in Figure 1 for the different methods evaluated. (**A**) NMF, (**B**) SNMF, (**C**) SSNMF, (**D**) 2S-NMPCF, (**E**) 1S-NMPCF, (**F**) T-NMPCF, (**G**) CNMF, (**H**) ST-NMPCF, (**I**) IIS${}_{0}$-NMPCF and (**J**) IIS-NMPCF.

**Figure 11:**
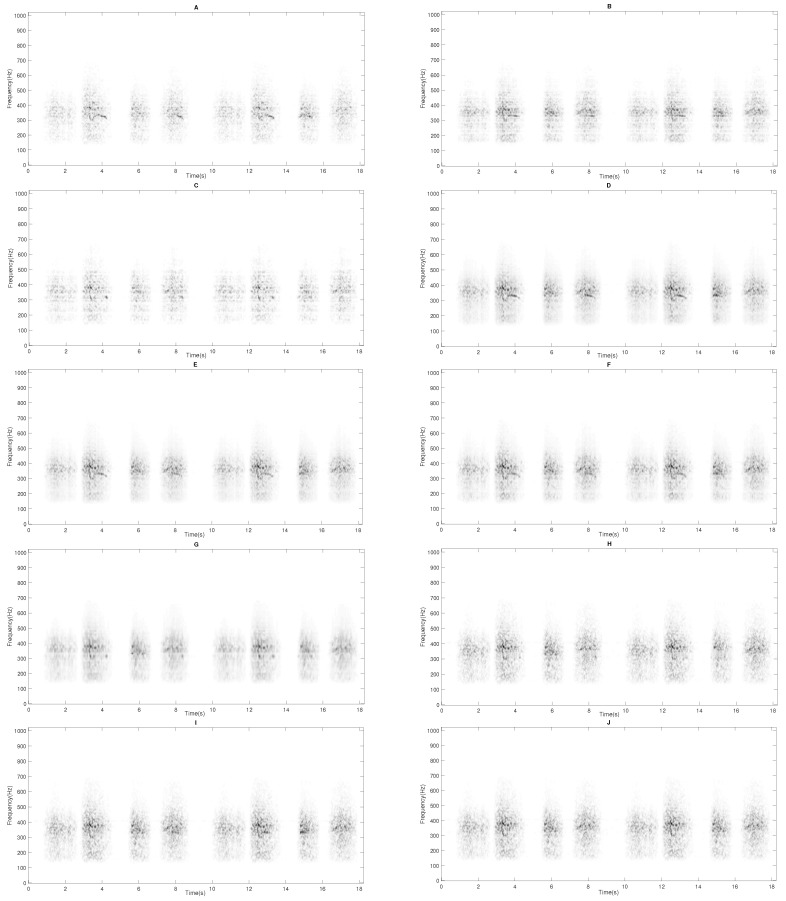
The estimated respiratory spectrogram ${\hat{X}}_{R}$ obtained from the input spectrogram X shown in Figure 1 for the different methods evaluated. (**A**) NMF, (**B**) SNMF, (**C**) SSNMF, (**D**) 2S-NMPCF, (**E**) 1S-NMPCF, (**F**) T-NMPCF, (**G**) CNMF, (**H**) ST-NMPCF, (**I**) IIS${}_{0}$-NMPCF and (**J**) IIS-NMPCF.

Finally, a set of spectrograms are presented in Figure 10 and Figure 11 in order to display the sound separation performance obtained by each of the assessed methods. Unlike the other evaluated methods, it can be observed that the proposed method IIS-NMPCF removes most of the RS in the estimated wheezing spectrogram ${\hat{X}}_{W}$ keeping most of the wheezing spectral content. This fact confirms the advantage of the proposed method since most of the clinical useful information contained in the estimated spectrogram ${\hat{X}}_{W}$ will be available to the physician to maximize the reliability of medical diagnosis. The MATLAB implementation of the proposed method is shared by the authors and can be downloaded from GitHub (https://github.com/JTORRECRUZ/Sensors_IIS-NMPCF).

## 5. Conclusions

We propose an extended version of Non-negative Matrix Partial Co-Factorization (NMPCF) approach to separate wheezing and respiratory sounds improving their acoustic quality. We assume that RS can be considered as sound events that are repeated during the human breathing process. However, WS may or may not be present along the segments due to the unpredictable nature of the pulmonary disorder. The main contribution of the proposed method is to add importance to the segments classified as non-wheezing to improve the sound separation performance of the conventional NMPCF which treats all segments of the input spectrogram equally. As a result, our proposal (IIS${}_{0}$-NMPCF/IIS-NMPCF) is able to characterize RS more accurately by forcing to model more on those non-wheezing segments in the bases sharing process into the NMPCF approach.

The main conclusions from the experimental results indicate that adding more importance to the non-wheezing segments into the decomposition procedure (NMPCF) models more accurate the spectro-temporal characteristics related to repetitive sound events of the mixture. In this work, these repetitive sound events are represented by RS that are present in all cycles of the breathing. Experimental SDR, SIR and SAR results report that the proposed method IIS-NMPCF outperforms significantly all evaluated methods providing competitive and promising results in the wheezing sound separation. This fact confirms the ability of the proposed method to improve the sound quality of WS maximizing both the removal of the acoustic interference caused by RS and that as much wheezing content is maintained. As a result, all useful medical information contained in the estimated wheezing can be clearly preserved.

It can be observed that the separation performance for the different evaluated methods drops when the SNR decreases. Considering the acoustic scenario in which RS are louder than WS (SNR < 0 dB), WS are barely audible due to the high interference produced by RS. Although in this case the reduction of the SNR implies a greater time-frequency overlapping from RS to WS, our proposal still achieves the best performance compared to the other baseline methods evaluating. Therefore, the proposed method can be considered an useful tool to be applied in sound environments in which WS are barely audible.

Future work will focus on the development of new constraints to be incorporated into NMF-based approaches for modelling different types of WS according to their spectral content in order to automatically classify the severity of the lung disorder. 

## Figures and Tables

**Table 1:** Characteristics of each database.

ID1	ID2	ID3	ID4	ID5	ID6	ID7	ID8	ID9
P1	48	5–24	721	[0–9]	[4–16]	496	[1–8]	92
T1H	16	7–22	251	5	[6–14]	126	[1–5]	41
T1M	16	7–22	251	0	[6–14]	126	[1–5]	41
T1L	16	7–22	251	−5	[6–14]	126	[1–5]	41

ID1: identifier; ID2: number of recordings captured from unhealthy subjects; ID3: the shortest and longest duration, in seconds, captured from recordings; ID4: total duration in seconds; ID5: the lowest and highest SNR, in dB, between WS and RS; ID6: the minimum and maximum number of respiratory events found in the recordings; ID7: the total number of respiratory events; ID8: the minimum and maximum number of wheezes found in the recordings; ID9: the total number of wheezes.

**Table 2:** The optimal parameters of the proposed method that obtain the best wheezing audio quality evaluating the dataset P1.

**IIS-NMPCF Approach Parameters**	$K_{W}$	$K_{R}$	$\lambda_{R}^{0}$	α	$\lambda_{R}^{1}$	$\lambda_{W}$
**Optimal values**	64	32	10	1	0.1	0.01

**Table 3:** Characteristics of the methods evaluated.

Method	Approach	Modelling Associated to WS and RS
NMF	NMF	
SSNMF	NMF	y[n]
SNMF	NMF	y[n] and z[n]
CNMF	NMF	Sparseness and Smoothness constraints
1S-NMPCF	NMPCF	y[n]
2S-NMPCF	NMPCF	y[n] and z[n]
T-NMPCF	NMPCF	*L*-segments
ST-NMPCF	NMPCF	*L*-segments and y[n]
IIS${}_{0}$-NMPCF	NMPCF	*L*-segments and $C^{\left(l\right)}$
IIS-NMPCF	NMPCF	*L*-segments, $C^{\left(l\right)}$ and y[n]

**Table 4:** Analysis of the statistical significance of the respiratory/wheezing SDR, SIR and SAR results comparing the proposed method (IIS-NMPCF) with the other evaluated methods using an one-sided paired *t*-test in the databases T1H (see Figure 7).

Method	SDR${}_{r}$	SIR${}_{r}$	SAR${}_{r}$	SDR${}_{w}$	SIR${}_{w}$	SAR${}_{w}$
NMF	$6.1\times 10^{-10}$	$4.1\times 10^{-10}$	$5.4\times 10^{-3}$	$1.9\times 10^{-11}$	$1.8\times 10^{-11}$	$4.8\times 10^{-7}$
SSNMF	$1.4\times 10^{-7}$	$5.5\times 10^{-8}$	$4.8\times 10^{-3}$	$3.2\times 10^{-10}$	$4.4\times 10^{-12}$	$8.9\times 10^{-8}$
SNMF	$1\times 10^{-7}$	$3.1\times 10^{-7}$	$4.5\times 10^{-8}$	$7.9\times 10^{-12}$	$1.4\times 10^{-10}$	$1.2\times 10^{-2}$
2S-NMPCF	$3.3\times 10^{-7}$	$5.5\times 10^{-6}$	$4.9\times 10^{-7}$	$2.6\times 10^{-11}$	$1.7\times 10^{-10}$	$1.8\times 10^{-3}$
1S-NMPCF	$6\times 10^{-6}$	$4\times 10^{-7}$	$2.7\times 10^{-6}$	$5.2\times 10^{-10}$	$9.2\times 10^{-11}$	$4.9\times 10^{-3}$
T-NMPCF	$3.8\times 10^{-5}$	$5.7\times 10^{-5}$	$3.3\times 10^{-4}$	$4.4\times 10^{-9}$	$8.9\times 10^{-9}$	$2.9\times 10^{-2}$
CNMF	$1.6\times 10^{-4}$	$1.7\times 10^{-3}$	$1.8\times 10^{-7}$	$2.6\times 10^{-6}$	$1.3\times 10^{-6}$	$7.2\times 10^{-4}$
ST-NMPCF	$1.5\times 10^{-4}$	$9.5\times 10^{-6}$	$5.2\times 10^{-2}$	$3.9\times 10^{-5}$	$5.3\times 10^{-7}$	$2.2\times 10^{-2}$
IIS${}_{0}$-NMPCF	$4\times 10^{-2}$	$2.2\times 10^{-3}$	$1\times 10^{-1}$	$4.2\times 10^{-2}$	$8.2\times 10^{-3}$	$1.1\times 10^{-1}$

Each cell shows the parameter ρ that represents the probability of setting a statistically significant result. Considering a confidence interval of 95%, small values of ρ < 0.05 indicate that there exists statistical significance of the results evaluated.

**Table 5:** Analysis of the statistical significance of the respiratory/wheezing SDR, SIR and SAR results comparing the proposed method (IIS-NMPCF) with the other evaluated methods using an one-sided paired *t*-test in the databases T1M (see Figure 8).

Method	SDR${}_{r}$	SIR${}_{r}$	SAR${}_{r}$	SDR${}_{w}$	SIR${}_{w}$	SAR${}_{w}$
NMF	$4\times 10^{-13}$	$4.1\times 10^{-14}$	$9.4\times 10^{-2}$	$6.2\times 10^{-13}$	$2\times 10^{-12}$	$2.7\times 10^{-9}$
SNMF	$4.3\times 10^{-13}$	$7.3\times 10^{-13}$	$4.3\times 10^{-2}$	$2.8\times 10^{-13}$	$1\times 10^{-10}$	$1.3\times 10^{-10}$
SSNMF	$9.7\times 10^{-11}$	$2.3\times 10^{-10}$	$2.5\times 10^{-6}$	$5.6\times 10^{-11}$	$4.3\times 10^{-10}$	$2.9\times 10^{-7}$
2S-NMPCF	$6.9\times 10^{-11}$	$1.1\times 10^{-11}$	$4.9\times 10^{-6}$	$5.1\times 10^{-11}$	$5.8\times 10^{-11}$	$3.5\times 10^{-8}$
1S-NMPCF	$8.7\times 10^{-8}$	$4.9\times 10^{-10}$	$1.3\times 10^{-6}$	$1.7\times 10^{-8}$	$1.4\times 10^{-9}$	$3.3\times 10^{-7}$
T-NMPCF	$9.7\times 10^{-9}$	$3.7\times 10^{-9}$	$1.1\times 10^{-7}$	$6.9\times 10^{-9}$	$1.6\times 10^{-7}$	$1.2\times 10^{-9}$
CNMF	$9.6\times 10^{-7}$	$1.1\times 10^{-5}$	$5.7\times 10^{-5}$	$9.4\times 10^{-6}$	$7.4\times 10^{-5}$	$5.2\times 10^{-7}$
ST-NMPCF	$1.9\times 10^{-4}$	$1.6\times 10^{-4}$	$4.4\times 10^{-2}$	$8.4\times 10^{-5}$	$4.3\times 10^{-4}$	$1.3\times 10^{-9}$
IIS${}_{0}$-NMPCF	$4.1\times 10^{-2}$	$6.3\times 10^{-4}$	$4\times 10^{-1}$	$2\times 10^{-2}$	$3.1\times 10^{-2}$	$3.3\times 10^{-4}$

Each cell shows the parameter ρ that represents the probability of setting a statistically significant result. Considering a confidence interval of 95%, small values of ρ < 0.05 indicate that there exists statistical significance of the results evaluated.

**Table 6:** Analysis of the statistical significance of the respiratory/wheezing SDR, SIR and SAR results comparing the proposed method (IIS-NMPCF) with the other evaluated methods using an one-sided paired *t*-test in the databases T1L (see Figure 9).

Method	SDR${}_{r}$	SIR${}_{r}$	SAR${}_{r}$	SDR${}_{w}$	SIR${}_{w}$	SAR${}_{w}$
NMF	$2.1\times 10^{-9}$	$6.8\times 10^{-12}$	$3.9\times 10^{-8}$	$2.2\times 10^{-9}$	$1.5\times 10^{-12}$	$8.6\times 10^{-10}$
SNMF	$5.5\times 10^{-10}$	$3\times 10^{-11}$	$1.9\times 10^{-5}$	$1.7\times 10^{-9}$	$2.7\times 10^{-12}$	$8\times 10^{-12}$
SSNMF	$5.3\times 10^{-10}$	$1.1\times 10^{-13}$	$6.5\times 10^{-10}$	$1.2\times 10^{-9}$	$6.2\times 10^{-11}$	$3.6\times 10^{-10}$
2S-NMPCF	$2.8\times 10^{-10}$	$3.1\times 10^{-9}$	$4.7\times 10^{-10}$	$1.1\times 10^{-8}$	$1.2\times 10^{-10}$	$9.3\times 10^{-10}$
1S-NMPCF	$1.8\times 10^{-9}$	$9.9\times 10^{-12}$	$5.5\times 10^{-12}$	$2.1\times 10^{-8}$	$3.3\times 10^{-9}$	$2.9\times 10^{-6}$
T-NMPCF	$1.5\times 10^{-7}$	$1.7\times 10^{-8}$	$5.4\times 10^{-12}$	$1.8\times 10^{-7}$	$1.2\times 10^{-7}$	$2.5\times 10^{-8}$
CNMF	$2\times 10^{-5}$	$4.4\times 10^{-4}$	$3.9\times 10^{-2}$	$4.7\times 10^{-9}$	$3.4\times 10^{-4}$	$5.6\times 10^{-6}$
ST-NMPCF	$5.6\times 10^{-4}$	$4\times 10^{-6}$	$3.7\times 10^{-4}$	$1.9\times 10^{-6}$	$1.1\times 10^{-3}$	$5.2\times 10^{-6}$
IIS${}_{0}$-NMPCF	$3.6\times 10^{-2}$	$9.6\times 10^{-3}$	$3\times 10^{-6}$	$2.5\times 10^{-3}$	$2.7\times 10^{-2}$	$4.3\times 10^{-3}$

Each cell shows the parameter ρ that represents the probability of setting a statistically significant result. Considering a confidence interval of 95%, small values of ρ < 0.05 indicate that there exists statistical significance of the results evaluated.
